# P-368. Long-range Air Dispersal as An Important Source of Environmental Contamination in *Candida auris* Clustering: Possible Infection Control Implication

**DOI:** 10.1093/ofid/ofae631.569

**Published:** 2025-01-29

**Authors:** Wing-Yu Fung, Ryan Yiu-Fai Cheng, Anthony Pak-Yuen Yau, Suet-Yi Lee, Ngan-Han Chan, Yuk-Shan Wong, Naomi Hua-Yin Cheng, Sally Cheuk-Ying Wong, David Christopher Lung

**Affiliations:** Department of Pathology, Queen Elizabeth Hospital, Hong Kong Special Administrative Region, HONG KONG, Hong Kong; Department of Respiratory Medicine, Kowloon Hospital, Hong Kong Special Administrative Region, Hong Kong, Not Applicable, Hong Kong; Department of General medicine, Bendigo Health, Australia, Hong Kong, Not Applicable, Hong Kong; Infection Control Team, Queen Elizabeth Hospital, Hong Kong Special Administrative Region, Hong Kong, Not Applicable, Hong Kong; Infection Control Team, Queen Elizabeth Hospital, Hong Kong Special Administrative Region, Hong Kong, Not Applicable, Hong Kong; Infection Control Team, Kowloon Hospital, Hong Kong Special Administrative Region, Hong Kong, Not Applicable, Hong Kong; Department of Pathology, Queen Elizabeth Hospital, Hong Kong Special Administrative Region, HONG KONG, Hong Kong; Department of Pathology, Queen Elizabeth Hospital, Hong Kong Special Administrative Region; Department of Pathology, Hong Kong Children’s Hospital, Hong Kong Special Administrative Region, Hong Kong, Not Applicable, Hong Kong; Department of Pathology, Queen Elizabeth Hospital, Hong Kong Special Administrative Region; Department of Pathology, Hong Kong Children’s Hospital, Hong Kong Special Administrative Region, Hong Kong, Not Applicable, Hong Kong

## Abstract

**Background:**

Long-range air dispersal of *Candida auris* has recently been described. Contact precautions alone may not be sufficient to target this mode of transmission. Hence, we study the factors affecting the extent of environmental contamination including air grilles in sporadic *C. auris* hospitalized patients, and in outbreak settings.

**Figure:**
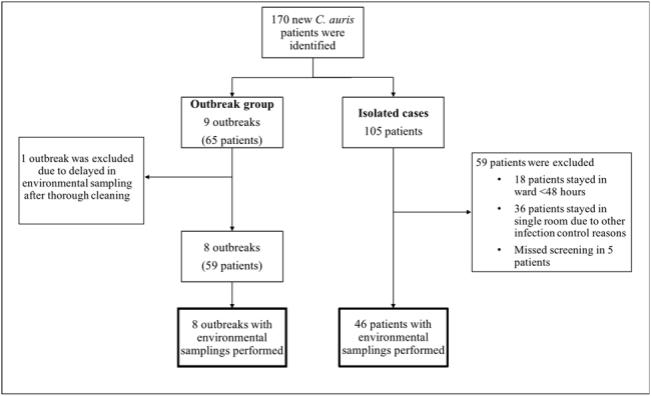

Study flow chart

**Methods:**

Outbreaks and sporadic *C. auris* cases involving two local hospitals from March to October 2023 were retrospectively analysed. *C. auris* screening was performed for patients with defined risk factors or regular surveillance as per hospital policy. Environmental samples were taken from high-touch surfaces and air grilles from patients’ environment. Correlation between scale of *C. auris* clustering and extent of environmental contamination was analysed.

**Figure:**
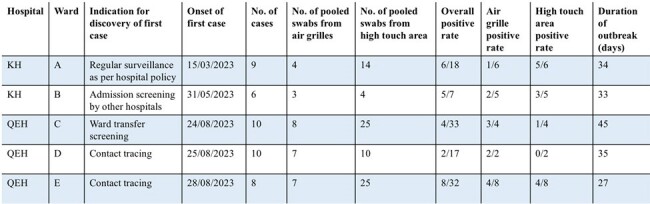

Outbreaks with positive environmental sampling

**Results:**

A total of 170 new *C. auris* patients were identified, including 65 patients from nine outbreaks and 105 sporadic cases. Environmental screening was performed in eight outbreaks and 46 sporadic isolated cases (Fig 1). Environmental contamination rate of the outbreak group was significantly higher than the sporadic group (15.1% vs 2.5%, P< 0.05). Among outbreak groups, longer median duration of outbreak (34 [27-45] vs 7 [7-12] days, p< 0.05), and more patients’ involvement (9 [6-10] vs 5 [4-7], p=0.05) were associated with higher environmental contamination rate (Table 1). Ward contamination was more frequently detected in sporadic patients with longer duration of stay, though it was not statistically significant (30.8% vs 10%, p=0.09). Air grille samples had a significantly higher contamination rate than high-touch surfaces in both outbreak (41.4% vs 16.7%, p< 0.05) and isolated group (19.7% vs 6.7%, p< 0.05).

**Figure:**
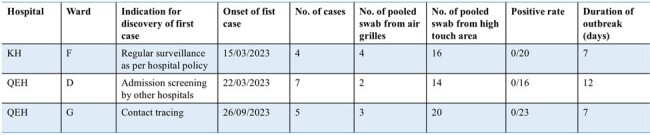

Outbreaks with negative environmental sampling

**Conclusion:**

Prolonged and sizeable *C. auris* clustering was associated with more extensive environmental contamination, particularly air grilles. Long-range air dispersal, as evidenced by air grille contamination, is a possible risk factor for propagation in outbreaks. Contact precautions together with physical segregation to limit air dispersal is recommended. Decontamination of air grille is necessary to prevent further spread. The role of air grille surveillance as a surrogate marker of ward contamination is worthy of further investigation.

**Disclosures:**

**All Authors**: No reported disclosures

